# Identification of Genes Important for Cutaneous Function Revealed by a Large Scale Reverse Genetic Screen in the Mouse

**DOI:** 10.1371/journal.pgen.1004705

**Published:** 2014-10-23

**Authors:** Tia DiTommaso, Lynelle K. Jones, Denny L. Cottle, The WTSI Mouse Genetics Program, Anna-Karin Gerdin, Valerie E. Vancollie, Fiona M. Watt, Ramiro Ramirez-Solis, Allan Bradley, Karen P. Steel, John P. Sundberg, Jacqueline K. White, Ian M. Smyth

**Affiliations:** 1 Department of Biochemistry and Molecular Biology, Monash University, Clayton, Melbourne, Australia; 2 Wellcome Trust Sanger Institute, Genome Campus, Hinxton, Cambridge, United Kingdom; 3 Centre for Stem Cells and Regenerative Medicine King's College London, Guy's Hospital, London, United Kingdom; 4 Wolfson Centre for Age-Related Diseases, King's College London, Guy's Campus, London, United Kingdom; 5 The Jackson Laboratory, Bar Harbor, Maine, United States of America; 6 Department of Anatomy and Developmental Biology, Monash University, Clayton, Melbourne, Australia; Stanford University School of Medicine, United States of America

## Abstract

The skin is a highly regenerative organ which plays critical roles in protecting the body and sensing its environment. Consequently, morbidity and mortality associated with skin defects represent a significant health issue. To identify genes important in skin development and homeostasis, we have applied a high throughput, multi-parameter phenotype screen to the conditional targeted mutant mice generated by the Wellcome Trust Sanger Institute's Mouse Genetics Project (Sanger-MGP). A total of 562 different mouse lines were subjected to a variety of tests assessing cutaneous expression, macroscopic clinical disease, histological change, hair follicle cycling, and aberrant marker expression. Cutaneous lesions were associated with mutations in 23 different genes. Many of these were not previously associated with skin disease in the organ (*Mysm1, Vangl1, Trpc4ap, Nom1, Sparc, Farp2*, and *Prkab1*), while others were ascribed new cutaneous functions on the basis of the screening approach (*Krt76, Lrig1, Myo5a, Nsun2*, and *Nf1*). The integration of these skin specific screening protocols into the Sanger-MGP primary phenotyping pipelines marks the largest reported reverse genetic screen undertaken in any organ and defines approaches to maximise the productivity of future projects of this nature, while flagging genes for further characterisation.

## Author Summary

Recent developments in high throughput applications to manipulate and inactivate specific genes in mouse embryonic stem cells (ES cells) have allowed for the initiation of large scale reverse genetic screens in the mouse. The immediate connection of a phenotype to a mutated (null) gene represents a paradigm shift in our ability to explore gene function. This study utilized such a screening approach to investigate the genetic contribution to skin development and homeostasis. Not only does this approach provide insight into the genetics of skin biology, it is also instrumental in generating novel models with which to study the genetic underpinnings of skin disease. Initial screening of 562 mutated genes in mice uncovered previously unrecognized genes involved in the biology of this organ and identified novel functions for previously studied genes associated with epidermal phenotypes. Taken together, these results highlight high throughput screening approaches that are valuable in reverse genetic screening and provide a pool of mouse mutants, available to the scientific community, that will serve as the basis for further detailed investigations into skin function and skin disease.

## Introduction

**Figure 1:**
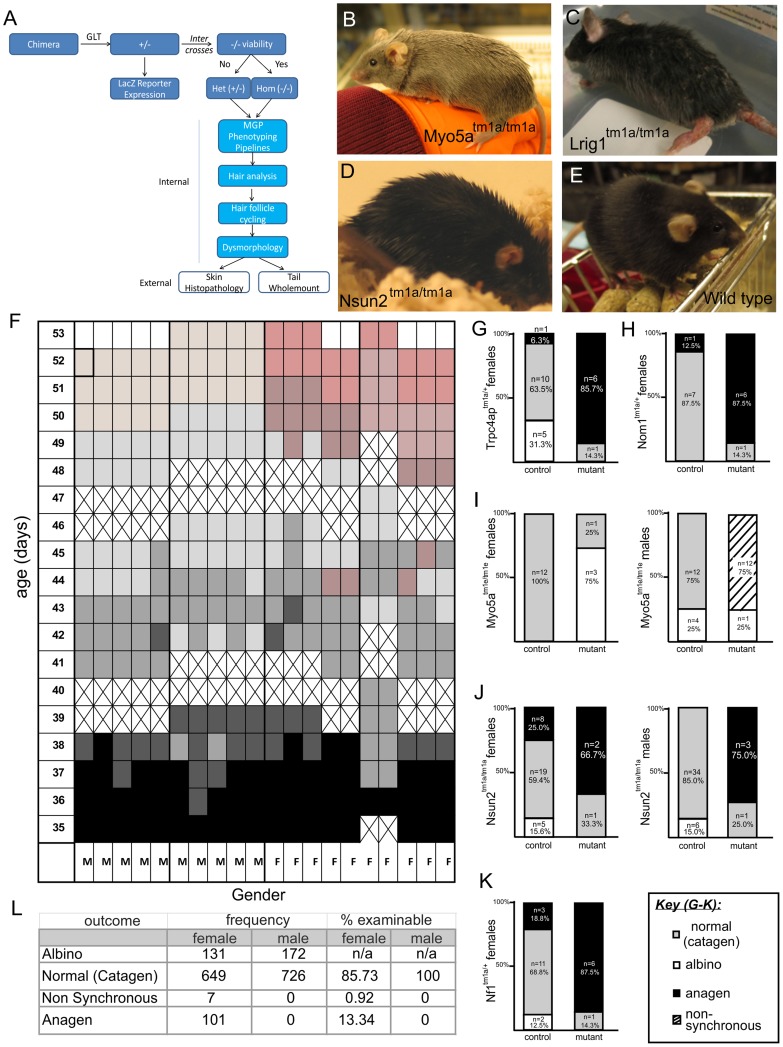
Overview of pipeline, hair follicle cycling baseline and phenotypes. A) Flow-chart illulstrating pipeline of skin phenotyping. B–E) Examples of obvious coat phenotypes in *Myo5a, Lrig1* and *Nsun2* strains relative to wild-type mice. F) 10 male and 10 female pigmented wild type mice were shaved and skin colour assessed from 35–53 days of age. Results are shown in a grid where cell color represents skin colour. Black indicates anagen, shades of grey (and dark pink) indicate catagen, pale pink indicates telogen for males, pink indicates telogen for females, and crosses indicate days mice were not assessed. Mutant mice and matched wild type controls were next shaved in weekly cohorts and dorsal skin assessed for hair cycle phase using the skin color method above at an age of 41–43 days. Pigmented mice were assessed as normal (grey skin/in catagen), non synchronous (mixed patches of hair cycle phases), and anagen (black skin). Albino mice could not be assessed and were excluded from the analysis. G–K) Shows *Nf1^tm1a^*; *Nsun2^tm1a^*; *Myo5a^tm1e^*; *Trpc4ap^tm1a^*; *Nom1^tm1a^* demonstrated signs of abnormally in constrast to their wild type controls. L) Table summarises wild type findings for baseline reference.

Recent advances in the high throughput production of targeted mutant mice using gene targeting in ES cells have made it possible to rapidly and effectively produce knockout mouse lines for use in phenotypic screening [1], [2], [3], [4]. Such approaches are now beginning to yield significant insights into mammalian gene function [1], [5]. Central to recent community efforts has been the Wellcome Trust Sanger Institute's Mouse Genetics Project (Sanger-MGP), which is undertaking a high throughput reverse genetic screen using the knockout ES cells generated by the International Knockout Mouse Consortium, which has now merged with the International Phenotyping Mouse Consortium (www.mousephenotype.org). To maximise the value of this resource, it is important to develop organ specific secondary screens to complement the broad phenotyping efforts applied in existing production pipelines. To this end, we implemented a multi-parameter, multi-test skin screen to identify mouse models of skin dysfunction and to elucidate genetic pathways important for skin development and function. In this study, we detail the skin assessment pipelines internal to the Sanger-MGP as well as skin analyses performed externally, with the exception of the fluorescent tail whole mount analysis, published elsewhere [6]. A flowchart for the pipeline is summarised in Figure 1A and presented in more detail in Figure S1A.

The skin is the largest of the intermediate sized organs in the human body [7] and it plays integral roles in immune surveillance, wound healing and protection from environmental challenge [8]. Skin related conditions account for significant health expenditure and top the list of reasons for general practice appointments in the U.S. [9]. A considerable number of skin conditions have genetic underpinnings [10]. Therefore, understanding the genetic basis of skin development and skin disorders has the potential to greatly impact both the diagnosis and treatment of skin related conditions. Moreover, a significant number of other systemic diseases or those principally affecting other organs also manifest themselves in the skin. Such conditions include diabetes mellitus, chronic kidney disease, celiac disease, and rheumatoid arthritis. As a highly regenerative organ in which developmental gene expression is often recapitulated during adulthood, the skin also provides a window into many of the programs central to differentiation and homeostasis. The skin and its associated “mini organ”, the hair follicle, are particularly attractive targets for such a screen because of their ready accessibility and dynamic nature [11]. Furthermore, the clinical pathology and physiology based mouse phenotyping “pipelines” are not designed to find subtle lesions affecting the skin [12]. For these reasons a skin specific phenotyping program was implemented on the genetically modified mice produced by the Sanger-MGP with a goal of defining new and previously unappreciated genetic determinants of skin disease.

In this paper mouse lines carrying mutations in each of 562 genes were investigated, and phenotypes were identified as a result of mutations in 23 genes (Table S1). These skin studies have identified genes not previously associated with skin phenotypes and ascribe further functions to genes which were previously associated with skin disease. We also used these tests to assign 25 *Mammalian Phenotype* (MP) ontology terms [13] (http://www.informatics.jax.org/searches/MP_form.shtml), 2 of which are novel (Table S1). The scale of the screen also provides the opportunity to assess the relative value of different phenotyping approaches, with a view to implementing these screens in other proposed and ongoing high throughput reverse genetic screens.

## Results and Discussion

### The Screen

#### The skin phenotyping screen

The vast majority of the mutant lines examined in this study (>95%) were derived from the EUCOMM/KOMP knockout first conditional-ready targeted ES cell resource on a C57BL/6N background [5]. However, to assess germline transmission mice were crossed with albino mice, homozygous for the recessive albino allele, C57BL/6J *Tyr^c-Brd^*. This strategy preserves the C57BL/6 genetic background while indicating germline transmission by simple black/white coat colour. In subsequent breeding this white coat phenotype was coincidentally inherited along with the targeted cassette. Full details of the genetic background of strains are presented in Table S1. These lines are predicted to produce null alleles as a consequence of the insertion of the strong *Engrailed-2* splice acceptor upstream of a *lacZ* reporter cassette followed by a SV40 polyA to terminate gene transcription and prevent potential splicing with downstream exons [14] (Figure S2). Primary screens were carried out either on viable homozygotes or, in the case of those alleles which exhibited embryonic lethality, on heterozygote animals (Table S1). Over the course of testing, mutant mice underwent visual tests to screen for obvious phenotypes (typically 7 male and 7 female mutants per line, Table S1, Figure 1A). A number of these were designed specifically to assess cutaneous phenotypes. As a requirement for other non-skin phenotyping pipelines performed in parallel to our skin screen, mice were fed on a high fat diet. The altered diet did not cause overt changes in skin phenotypes. The appearance of the coat was initially assessed at 4 weeks of age, prior to the implementation of the high fat diet, then a detailed visual assessment of the skin, nail units, pelage, and teeth was performed at 10 weeks of age. At the time of necropsy (16 weeks of age) dorsal skin was collected for histology and immunohistochemical assays. Hair follicle cycling defects were evaluated at 6 weeks of age by shaving a patch of hair at the dorsal thoraco-lumbar region. We found this time point to coincide with completion of the synchronous, second postnatal anagen phase of the hair cycle and catagen transition into telogen [15]. The hair analysis and hair follicle cycling screens were included as a part of the internal primary pipeline at the Sanger-MGP, whereas the screen for skin histopathology was conducted externally. We grouped the characterisation into three screens (Hair analysis and visual dysmorphology; Hair follicle cycling; Skin histopathology) and used them to examine 562 unique gene knockouts. We identified 23 genes (4.1%) with 25 putative phenotypes (Table S1) related to skin biology. One such gene identified from the screen, Keratin 76, is characterised in depth in an accompanying manuscript in this issue of PLoS Genetics [16]. The results of each of these specific screens are discussed below.

#### Hair analysis and visual dysmorphology screening

The simplest aspect of the skin screen comprises the visual assay for overt abnormalities of the pelage (Figure 1B–E). This key primary screen was performed in the Sanger-MGP pipeline. Parameters relevant to skin in this screen include pelage appearance and coloration, skin and footpad pigmentation (excluding albino mice), facial vibrissae morphology, and length, morphology and number of nail units and teeth. Of the 263 phenotype parameters assessed in the Sanger-MGP pipeline (comprising 147 categorical and 116 continuous measurements), 43 (16.4%) were directly related to skin and hair [1](Table S2). In addition, 362 different genes were screened identifying 23 that exhibited abnormal phenotypes in one or more skin-related organ systems (Table S1). Of these hits, 9 were directly related to skin and hair descriptors (Table 1) making skin defects the most common gross morphological finding together with craniofacial defects (n = 9) and defects in the eye (n = 4), tail/limbs (n = 2), and genitalia (n = 2). Of the 9 lines that exhibited a cutaneous phenotype, the most common reported phenotype was abnormal pigmentation (n = 5/9; *Krt76*, *Myo5a* (Figure 1B), *Mysm1*, *Vangl1*, *Sparc*) followed by general integument phenotypes (scruffy coat, greasy coat, scaly skin, hair loss n = 4/9, *Krt76*, *Lrig1* (Figure 1C), *Myo6*, *Nsun2* (Figure 1D)) and nail phenotypes (n = 1/9 *Prkab1*) (Table 1). A wild type mouse is shown in Figure 1E for comparison. We also examined the expression patterns of these 9 genes utilising the activity of the integrated beta galactosidase (*lacZ*) reporter gene and found that with the exception of two genes (*Myo5a* and *Prkab1*) all were expressed in the epidermis or dermis (Table 1, see later figures and Figure S3 for examples of specific genes). *Sparc lacZ* expression in tail whole mounts was ubiquitous (https://www.mousephenotype.org/data/genes/MGI:98373). *Myo6* expression in ear epithelia has been reported using IHC [17]. While *Myo5a* and *Prkab1* expression were not detected via the *lacZ* reporter epidermal expression of *Myo5a* has been previously reported [18], and Prkab1 has wide ranging tissue expression [19], suggesting a *lacZ* reporter sensitivity issue.

**Table 1:** Genes identified in the primary phenotypic screen with cutaneous defects.

Gene	Protein	Expression	Biological Function	Skin Function	Primary Phenotypic Features	Skin Associated MP Terms
*Krt76*	Keratin 76	palate, paw pad, oral epithelium, fore stomach	intermediate filament, cytoskeleton, structural molecule activity	palatal keratin	behavior, integument	MP:0001510 - abnormal coat appearance MP:0010179 - rough coat MP:0000416 - sparse hair MP:0000575 - dark foot pads
*Lrig1*	leucine-rich repeats and immunoglobulin-like domains 1	most tissues including skin	Integral to membrane, tumor suppressor	Marker of hair follicle junctional zone stem cells	adipose, growth/size, hearing/vestibular/ear, homeostasis, immune, integument, limbs/digits/tail, skeleton, vision/eye	MP:0001191 - abnormal skin condition MP:0001192 - scaly skin
*Myo5a*	Myosin VA	brain, cartilage, pituitary gland, spinal cord, urinary system	ATP-dependant motor protein, actin filament based movement	melanocyte differentiation, pigmentation	integument, mortality/aging, pigmentation	MP:0000371 - abnormal coat/hair pigmentationMP:0002075 - diluted coat color
*Myo6*	Myosin V1	cochlear hair cells*	sensory perception of sound, auditory receptor cell differentiation, signal transduction	unknown	behavior, integument, skeletal, homeostasis/metabolism, immune	MP:0000367 - abnormal coat/hair morphology MP:0000418 - localized hair loss
*Mysm1*	Histone H2A deubiquitinase	most tissues including skin	histone deubiquitination, postivie regulator of transcription	unknown	adipose, behavior, cellular, craniofacial, growth/size, hematopoietic, homeostasis, immune, integument, limbs/digits/tail, mortality/aging, other, pigmentation, skeleton, vision/eye	MP:0000373 - belly spot MP:0000574 - abnormal foot pad morphology MP:0000575 - dark foot pads
*Nsun2*	NOL1/NOP2/Sun domain family member 2	ubiquitous	RNA methyltransferase, cell cycle, cell division	Hair follicle stem cell self-renewal	adipose, behavior, craniofacial, growth/size, hematopoietic, homeostasis, integument, limbs/digits/tail, reproductive, skeleton, vision/eye	MP:0001510 - abnormal coat appearance,
*Prkab1*	protein kinase, AMP-activated, beta 1 non-catalytic subunit	brain, eye, liver, skull	fatty acid biosynthetic process, fatty acid metabolic process, lipid metabolic process	unknown	resistance to diet induced obesity, liver steatosis, and hyperinsulinemia	MP:0000579 - abnormal nail morphology
*Sparc*	secreted acidic cysteine rich glycoprotein	ubiquitous	bone and lung development, cell migration, tissue remodelling	unknown	craniofacial, skeleton, vision/eye	MP:0010096 - abnormal incisor color
*Vangl1*	vang-like protein 1	skin, brain, cartilage, colon, bladder, eye, kidney, bone, thyroid, intestine, heart, lung, oesophagus, parathyroid, spinal cord	development, integral to membrane, protein binding	unknown	hematopoietic system, homeostasis/metabolism, integument	MP:0003849 - greasy coat, MP:0000574 - abnormal foot pad morphology, MP:0000575 - dark foot pads

#### Hair follicle cycling

The hair follicle is a dynamic mini organ that continually undergoes a controlled program of growth (anagen), regression (catagen), and resting (telogen). Accordingly, hair follicle morphogenesis is maintained by a complex program of proliferation, differentiation and death. Regulators of this process include developmentally important proteins such as members of the bone morphogenetic protein (BMP), hedgehog (HH) and wingless-related MMTV integration site (WNT) signalling pathways, as well as a variety of cytokines, transcription factors, and adhesion molecules (reviewed in [20]). A simple way to examine the stage of hair follicle cycle at a gross level is to assess skin color in shaved animals; as follicles in anagen darken the skin due to melanocyte activity [21]. This process is non-invasive and requires minimal expertise, making it highly suitable for application to high throughput screens. In newborn mice, the dorsal skin follicles of the animal undergo two synchronous cycles of growth and regression after birth [22], [23]. This synchronicity affords a unique opportunity to study the factors driving this process. Skin in anagen appears black, while skin in telogen appears pink. At approximately 6 weeks of age, hair follicles progress through the catagen regression stage of the cycle over 3 days [24]. This process correlates with a change in skin colour from the black of anagen towards the pink skin of telogen, giving catagen skin a distinctive intermediate dark pink appearance.

To generate a baseline dataset using the predominant background strain of the mice used in the screen, we examined a cohort of C57BL/6NTac mice (n = 10 females, 10 males) and assessed the colour of a shaved patch of skin daily for 3 weeks from 35 days post-partum. As expected, initially all mice were in the second postnatal anagen phase (and hence were black skinned) and exited anagen by ∼42 days of age, reaching telogen by 7–8 weeks of age. Males in this cohort reached telogen slightly ahead of females and generally had a more pale pink skin colour than females in telogen (Figure 1F). This pilot generated a reference baseline for hair cycling in wild type mice. Using a catagen time point of 41–43 days, mutant animals (typically 7 per sex per strain) were assessed to detect if the hair cycle was changed in any cohort of mice. Additionally 7 wild type controls were assessed to control for natural litter to litter variation. A total of 362 mutant mouse lines were analysed (Table S1). If the skin appeared black, it indicated either premature entry into or delayed exit from anagen, while if the skin appeared pink, it indicated that the hair cycle was accelerated and telogen initiated prematurely.

When comparing mutant and wild type hair cycling we identified 5 genotypes (5/362, 1.4%) with abnormalities in only one or both sexes: *Trpc4ap/+* (Figure 1G, also see Figure S3F for LacZ staining), *Nom1/+* (Figure 1H), *Myo5a*/*Myo5a* (Figure 1I), *Nsun2/Nsun2* (Figure 1J), and *Nf1/+* (Figure 1K, also see Figure S3C for LacZ staining). With the exception of *Nsun2*, none of these genes had previously been reported to influence hair follicle cycling. Some mutant strains could not be assessed in this assay due to complete penetrance of the co-inherited albino phenotype. As a consequence our screen most likely underestimates the incidence of these defects. We found two distinct types of cycling defects - a premature entry or delayed exit from anagen in which the skin remained black (*Trpc4ap* (Figure 1G), *Nom1* (Figure 1H), *Nsun2* (Figure 1J), *Nf1* (Figure 1K)) and a non-synchronous hair cycling phenotype in which patches of the coat were at different stages of the follicle cycle (*Myo5a*, Figure 1I). We did not observe any mutants with hair cycles that had accelerated entry into telogen. Interestingly, a number of these lines were heterozygotes, further illustrating the value in screening such animals for those lines which are homozygous lethal. Through the continued analysis of wild type control animals (a total of 1483 non-albino control mice (n = 757 females, n = 726 males)), we also confirmed a small but significant sex difference in cycling such that 14.26% (13.34% Anagen+0.92% Non synchronous) of female animals were not in catagen compared to males at this time point (Figure 1L, Chi-square test Yates p value = 0.023).

#### Skin histopathology and marker screening

**Figure 2:**
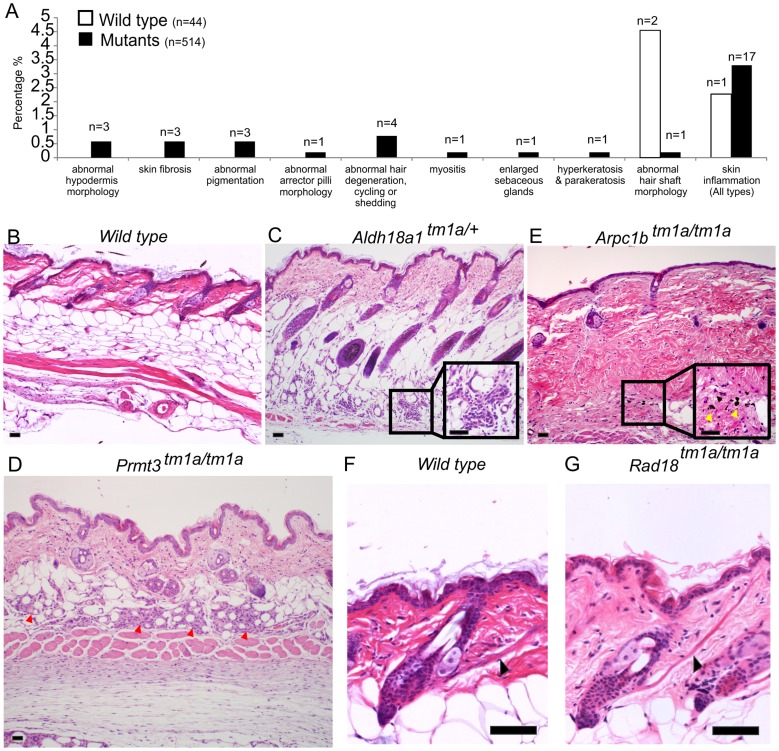
Skin histopathology overview. (A) The dorsal skin of 44 wild type mice and 514 mutant mice were assessed by an expert dermatologist. 3 wild type mice and 30 targeted alleles (in 30 mutant strains) showed abnormalities in one or more of the phenotypic categories listed (35 abnormalities total). The phenotypes of abnormal hair shaft morphology and skin inflammation were observed in both wild type and mutants and taken to be a background phenotype, therefore were not considered significant in the overall phenotypic analysis. Wild type images for comparison are shown in (B) and (F). Phenotypes unique to mutant animals included abnormal hypodermis dermis morphology (C,D), abnormal pigmentation (E) and abnormally prominent arrector pilli muscle (G). More specifically, *Aldh18a1^tm1a^/+* mice presented with mild, multifocal mixed inflammatory cell infiltration with mild fibrosis and distortion of adipocytes in the hypodermal fat layer potentially indicating abnormalities in white fat cells (C). *Prmt3^tm1a/tm1a^* mutant mice exhibited granulomatous steatitis (red arrowheads) (D). *Arpc1b^tm1a/tm1a^* mutant mice had mild to moderate multifocal areas of dermal fibrosis with pigment laden macrophages (yellow arrows) suggesting hair follicle rupture (E). *Rad18^tm1a/tm1a^* mutant mice had normal skin but unusually prominent arrector pili muscles (black arrows) (G). Scale bars are 50 µm.

An additional layer of screening was used to ascertain subtle phenotypes which might have been missed in other pipelines. This screen was undertaken at the point of terminal necropsy (age 16 weeks) at which point dorsal skin from 1 to 2 female mice was collected per mutant strain for histopathology assessment. Males were generally not assessed due to a higher incidence of fighting that could result in skin damage. Tissue sections from the mutant mouse lines and control mice were stained with H&E and underwent blinded assessment by an expert pathologist. Abnormalities were then re-classified into MP terms. For an abnormality to be scored as positive, presentation had to be multi-focal or diffuse, thus rare focal events were eliminated and considered part of the normal spectrum. Forty-four wild type control mice and 514 mutant animals (558 mice in total) were examined; this encompassed 512 mutant lines with a range of 1–2 mutant mice per line. Three wild type mice and 30 mutant animals (from 30 strains) demonstrated abnormalities and in some cases, multiple abnormalities, giving 35 phenotypes. Strain-related background phenotypes were identified in wild type mice in the MP term categories of abnormal hair shaft morphology (2 mice, 4.5%), and skin inflammation (1 mouse, 2.3%). Mutant animals also demonstrated these defects, for example, skin inflammation was detected in 17 mutants out of 514 (∼3.3%). Because of their presentation in wild type and test animals these phenotypes were not considered further, although it is possible that a fraction of the flagged mutant phenotypes are directly related to the induced genetic lesion. Further studies of individual lines would be required to establish this. However, the results of this histological analysis flagged other phenotypes not detected in wild type animals including 12 unique abnormalities/MP terms from 30 strains (Figure 2A and Table S1). These included altered hypodermal fat morphology in 3 lines (*Aldh18a1* (Figure 2C, see Figure S3A), *Ccdc57* and *Prmt3* (Figure 2D)), abnormal pigmentation in 3 lines (*Arpc1b* (Figure 2E), *Krt76* and *Prkcz* (Figure S3B)), abnormal muscle in 2 lines (*Scn3b*, myositis of panniculus carnosus, and *Rad18*, prominent arrector pili (Figure 2G, Figure S3E)), enlarged sebaceous glands (*Kdm4c*), hyperkeratosis (*Krt76*) and aberrant hair follicles or cycling (*Pfkl1, Nsun2, Farp2, Lrig1*; Figure S3D). The full histopathology dataset is included as Table S1.

### Case studies

A number of the screens identified new players in cutaneous biology or identified new phenotypes not previously associated with these genes. Some individual examples are discussed.

#### Pigmentation

**Figure 3:**
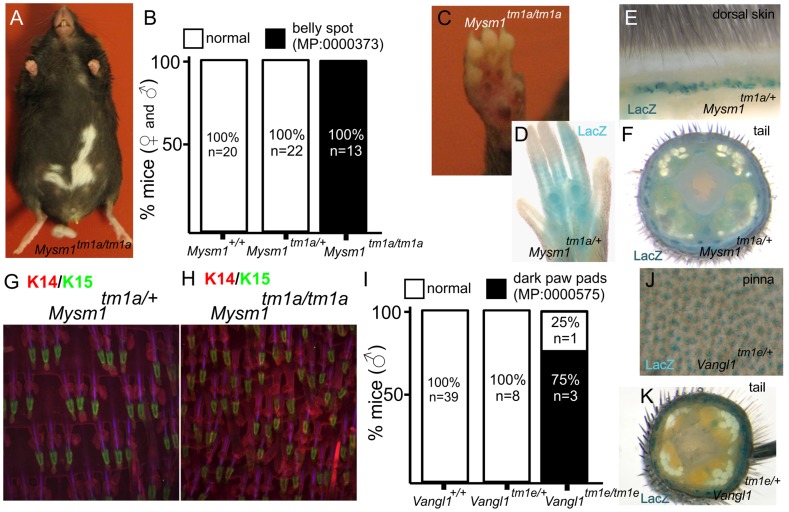
Examples of pigmentation phenotypes and expression patterns in genes with novel roles in skin biology. *Mysm1^tm1a/tm1a^* mice exhibited a range of pigmentation defects, including belly spots, and foot pad hyper-pigmentation (A, B, C). *LacZ* reporter expression of *Mysm1* is detected in the paw pad (D), dorsal skin and tail (E,F) whole mounts. Tail whole mount labelled with Keratin 14 (KRT14, red) and Keratin 15 (KRT15, green) indicate defects in hair follicle organization and associated structures in *Mysm1* knockouts (H) compared to heterozygotes (G). I) Analysis of footpad pigmentation in male *Vangl1^tm1a/tm1a^* mice. J,K) *LacZ* reporter expression of *Vangl1* in the ear skin (pinna) and tail whole mounts.

Pigmentation defects were one of the principal phenotypes reported (Table 1). Mice carrying mutations in genes responsible for melanocyte migration or the melanin production/transport machinery have been instrumental in establishing the field of mouse genetics. The efforts of the “mouse fancy” in the late 19^th^ century and the pioneering work of Cuenot and Castle in the early 20^th^ century utilised coat colour to first demonstrate Mendelian inheritance in mammals [25], [26]. Given the considerable period of time which has since passed, it is remarkable then that our screen uncovered new genes and phenotypes associated with the biology of the melanocyte. The first of these is *Mysm1*, which encodes a histone 2A deubiquitinase that regulates stabilisation of linker histone H1 with nucleosomes [27] and which is required for bone marrow stem cell function and haematopoiesis [28]. *Mysm1* mutant mice exhibited a white belly patch (Figure 3A,B) and there were also defects apparent in the pigmentation of the foot pad (Figure 3C). Foot pads normally have very little pigmentation, but there is an emerging family of genes whose disruption results in their hyperpigmentation [29], [30], [31]. Beta-galactosidase (*lacZ*) reporter activity indicates that *Mysm1* is expressed in the footpad (Figure 3D) and hair follicles of the dorsal coat and tail, principally in the lower region of the hair follicle (Figure 3E,F), a structure which is associated with a resident population of epidermal stem cells [32] and melanocytes. Additional parallel studies indicated that *Mysm1* is required for the normal patterning of hair follicles and sebaceous glands in the tail epidermis (compare heterozygous animal, Figure 3G, with homozygous mouse, Figure 2H [6]). Taken together, two distinct and previously undescribed manifestations of cutaneous *Mysm1* function are reflected in defects in pigmentation and patterning respectively. While the mechanism by which *Mysm1* acts to regulate these phenotypes is unclear, its known functions do draw attention to potential roles for histone modification and stem cell niche activation in regulating these processes. Belly spotting mutants of this type have long been recognised in mutant mouse strains (for example as a consequence of mutations in *Endrb, Kit, Mitf, Dock7, Pax3* [33], [34], [35], [36], [37]) but *Mysm1* differs to other known pigment-regulating genes in that it is an epigenetic regulator.

Pigmentation differences were also noted in mice carrying mutations in *Vangl1*. VANGL1 is known to be involved in the planar cell polarity pathway [38] and mutations in *Vangl1* have been linked to neural tube defects [39]. A heterologous protein, VANGL2, is important for establishing hair cell orientation in the inner ear [40]. Very few homozygous *Vangl1* knockouts survive to weaning (2% of live births from *Vangl1^tm1e^/+* intercrosses). However, in the small number of surviving adult knockouts generated, we observed hyperpigmentation of the paw pads specifically in homozygous male animals (3/4 homozygotes) but not in females (0/3 homozygotes) (Figure 3I). Given the low animal numbers for this semi-lethal line we cannot discriminate between variable penetrance of the phenotype and a *bona fide* sexual dimorphism. However, as no pigmentation defects have previously been associated with loss of *Vangl1* function our study therefore adds a new member to the family of genes affecting cutaneous pigmentation. Consistent with these observations we observe extensive expression of *Vangl1* in tail skin and the hair cells of the ear (Figure 3J,K).

#### Exogen

**Figure 4:**
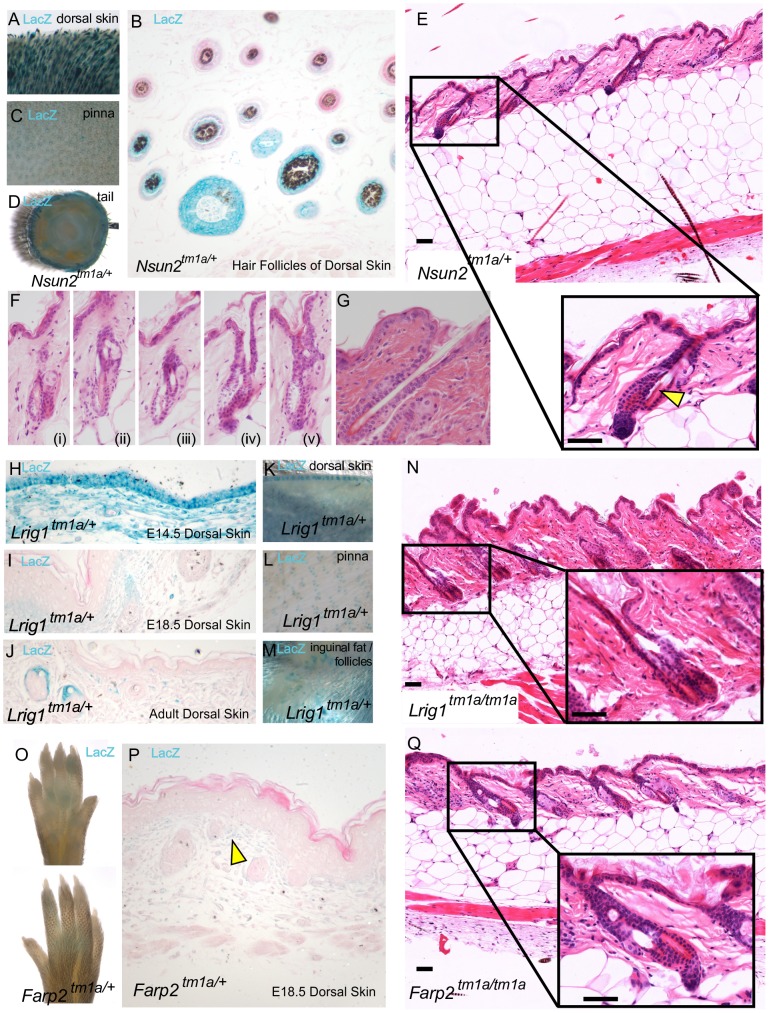
High throughput screening provides insights into molecular mechanisms of exogen in hair follicle cycling. *Nsun2* expression was reported by *lacZ* staining in *Nsun2^tm1a^/+* mice in the hair follicles of murine dorsal skin (A,B), the outer ear pinna (C), and tail (D). *Nsun2* mice demonstrated evidence of premature separation of the club hair from the surrounding follicle, leaving an empty area or breakage (E see arrowhead, Fi-v) in contrast to a normal follicle (G). *Lrig1* expression was reported by *lacZ* staining in *Lrig1^tm1a/+^* mice in the developing epidermis and dermis of E14.5 skin (H), the developing hair follicle and upper dermis of E18.5 skin (I), the upper dermis and junctional zone above sebaceous glands in adult skin (J), the hair follicles of adult murine dorsal skin (K), the outer ear pinna (L) and inguinal fat pads (M). *Lrig1* mice also demonstrate premature separation of the club hair (N). *Farp2* expression was reported by weak *lacZ* staining in footpads and dermis of *Farp2^tm1a/+^* mice (O,P see arrowhead). *Farp2* mice also demonstrated premature separation of the club hair (Q). Scale bars are 50 µm.

Exogen describes the stage of the hair cycle during which club hair fibres are shed from the hair follicles, incorporating both the actual loss and the molecular events which promote this [41]. Of the four genes identified in our histopathology screen that showed hair follicle cycling defects (Figure 2A), three were exogen specific; *Nsun2*, *Lrig1*, and *Farp2* and none of them have previously been associated with this process. *Nsun2*, (also known as MYC induced SUN domain containing protein 2 (*Misu2*)), is a direct downstream target of MYC in the skin and is essential for keratinocyte proliferation [42]. Mutations in *Nsun2* are known to affect the entry of hair follicle stem cells into the regenerative program and in regulating epidermal stem cell self-renewal and differentiation [43]. In agreement we observed *Nsun2* expression specifically in the hair follicles of dorsal skin (Figure 4A,B), ear (pinna-Figure 4C) and tail skin (Figure 4D). This gene was initially flagged in our screen as a result of coat abnormalities identified during primary visual assessments (Table 1) and as a factor in regulating hair follicle cycling (Figure 1J). Exogen defects in *Nsun2/+* mice can be seen as a detachment of the hair follicle from the bulge in Figure 4E (insert arrowhead) with additional examples shown in Figure 4Fi-v. Contrast these to a normal follicle which shows no detachment of the hair shaft within the hair follicle (Figure 4G). While we could not assess exogen in *Nsun2/Nsun2* mice (as these mice were in anagen at this time point), their scruffy coat (Figure 1D) and reported alopecia [43], may also be linked to this exogen defect.

Like *Nsun2*, *Lrig1* is also a MYC target gene [44] and it similarly helps to maintain the proliferative capacity of keratinocyte stem cells [45]. *LacZ* reporter studies highlight *Lrig1* expression initially in the epidermis and dermis of E14.5 skin (Figure 4H), which contracts in range to upper dermal cells and to hair follicles at E18.5 (Figure 4I), and the junctional zone above the sebaceous glands in adult dorsal skin (Figure 4J,K). Furthermore, Lrig1 *lacZ* reporter expression is detected in ear skin (pinna) (Figure 4L) and the hypodermal fat surrounding the follicles (Figure 4M). In this context, recent studies have implicated intradermal adipocyte precursors in follicular stem cell activation through PDGF signalling [46] as part of an increasing recognition of the influence of extracutaneous adipocytes on the hair cycle [46]. *Lrig1* mice also showed signs of hair follicle shedding (Figure 4N) which may explain the scruffy coat observed in this mice (Figure 1C).

The third gene, *Farp2*, is a guanine exchange factor known to be involved in osteoclast podosome dynamics [47] and axonal repulsion [48]. We detected *lacZ* signal for *Farp2* expression in the paw pad skin (Figure 4O) and weak dermal expression in E18.5 dorsal skin (Figure 4P, see arrowhead), which showed an exogen defect (Figure 4Q). Unlike *Nsun2* and *Lrig1*, there have been no direct reports of FARP2 interacting with MYC signalling or hair follicle cycling; however, it has been found to be a specific activator of RAC1 signalling, a proposed regulator of MYC activity in the skin [49]. Although none of these genes change significantly in expression as the club fibre nears release at the end of exogen [50] it could be that they (or MYC signalling generally) regulate this process through downstream signalling or by establishing a cellular phenotype that affects the transition to exogen. The signalling and adhesion factors mediating the active exogen process are considered to be regulated independently of normal hair follicle cycling [51], potentially explaining why we did not see defects in our hair cycling screen in the *Lrig1/Lrig1, Nsun2/+*, and *Farp2/Farp2* lines. This incidence of exogen defects is also likely under-estimated, as the majority of mice analysed at 16 weeks in our screen were in anagen.

### Screen productivity

**Figure 5:**
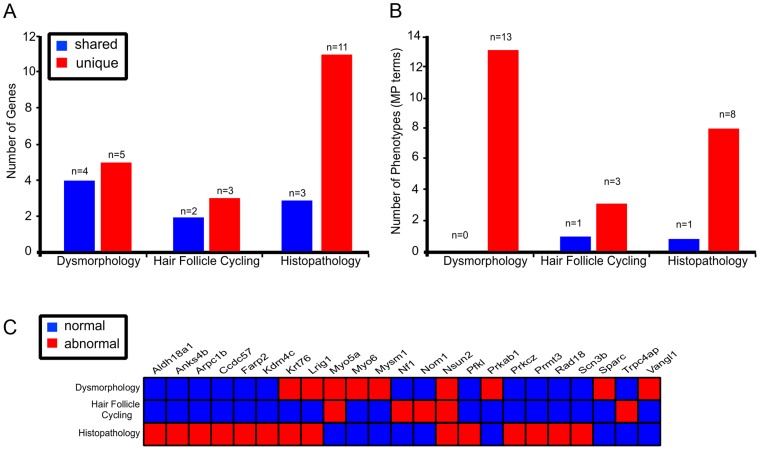
Multi parameter, multi test, organ specific screens add to the number of genes identified in skin biology. Each of the 3 different tests identified genes that were represented in other screens (shared) as well as genes that were unique to the test (A). Histopathology screen identified the highest number of unique genes flagged with 79% (n = 11/14) of genes not represented in any other skin screen. Different screens highlighted 25 unique MP terms, with only 1 MP term represented in multiple tests (B). Twenty three unique genes were identified across the 3 different skin tests with 4 represented in multiple tests (C).

A critical question for any screening approach is the relative value it brings in respect of identifying new phenotypes. While all parts of the skin screen identified mutant lines that were also identified in other tests, there were still sets of genes that were unique to each specific test(Figure 5A). For example, three out of the five genes identified in the hair follicle cycling screens were not identified using any other screening approach in our skin phenotyping pipeline (Figure 5A). This is also particularly true of the histopathology screen in which 11 of the 14 positive genotypes recorded no other hits in other skin pipeline – although the depth of screening by this approach was limited to 1–2 mice per line (Figure 5A). We also noted that each test identified MP terms that were not described by any other tests (Figure 5B). That is to say, the addition of extra screens for subtle phenotypes that were not reflected in gross changes to appearance were reliably able to identify both new genes and new phenotypes (Figure 5A,B,C; Table S1). Taken together this strongly argues for the inclusion of these more involved screens to flag skin related abnormalities, particularly in light of the relatively low hit rates achieved using simple dysmorphology. The screen in its current form may also underestimate the frequency of abnormal phenotypes due to the limitations imposed by sample size, albinism in strains, the stage of the hair cycle the necropsy samples, and a consequence of homozygous embryonic lethality. More detailed screens employing larger numbers of mice, together with skin-specific conditional sub-strains to overcome problems of complete knock-out lethality, will be required to accurately define a greater number and range of phenotypes.

### Observations

We detail one of the most comprehensive reverse genetic skin screens yet reported in mice. The gene targeting strategy, combined with a robust skin phenotyping protocol, has allowed immediate establishment of a preliminary connection between a genotype and a phenotype. Such approaches have only become feasible through the recent advent of high throughput, targeted mouse mutagenesis screens. Clearly, more detailed follow-up studies are necessary to elaborate on these initial findings and to investigate in greater detail the contribution of a given gene to the biology of the skin. One such follow-up study on *Krt76* is provided in an accompanying manuscript in this issue of PLoS Genetics [16]. Nonetheless high throughput approaches of this type will be central to flagging genes for further study. An argument might be made that molecules important to something as visually evident as skin development and homeostasis would already have been identified through mutagenesis approaches, or historically as a result of sporadically occurring mutations. However, the identification of two new genes that cause defects in pigmentation (*Mysm1* and *Vangl1*) and a further additional pigmentation phenotype in *Myo5a* mutants shows that even for the most obvious cutaneous feature, there remains considerable value in ongoing screening for new causative mutations. In particular, clinical and histological presentations vary between alleles based on the type of mutation (null, hypomorph) or inbred strain background (strain specific modifier genes), and so comparison between these “knockout ready” targeted mouse lines and existing spontaneous mutants present in various public repositories will be particularly informative in respect of gene function.

Similarly the skin might be considered an “easy” target for phenotypic screens, and that important genes could simply be identified based on a casual observation of the mutant animal in question. Our results argue that this is not the case. Instead we find that there is significant value in including secondary, organ specific screens in large scale projects of this nature. In deciding which phenotypic screens to apply to a given mutant mouse population one has to consider the likelihood that a given test will identify a phenotype not also apparent in other primary phenotyping screens. The object of such an assessment is therefore to reduce the costs and processes associated in examining such large cohorts of mice, whilst maximising the value of the resource (i.e. in robust phenotypes flagged). Interestingly, based on our experience, each of the phenotypic screens utilised added significantly to the base level of hits in the screen (Figure 5). While gross skin abnormality assessments shared the greatest overlap with the other screens employed, there were considerable numbers of genes identified in the more subtle screens that did not result in an obvious, visually assessable, phenotype in the mouse. The hair follicle cycling screen is a case in point. To all intents and purposes the visual phenotype of those animals which display abnormal hair follicle cycling is normal. However, a simple estimation of cycling stage uncovered a further class of mutants whose involvement in this process was never previously appreciated. The same is true of the section based histopathology screen, which was able to identify subtle but potentially valuable phenotypes. In both cases, whether through the subtle temporal regulation of follicle cycling or via the cellular diversity assessed in the pathology screen, looking a little deeper, can be a particularly valuable exercise. Taken together, our screen has identified a diverse collection of genes which will form the basis of further studies aimed at elucidating gene function in more detail. They also highlight the value of including detailed skin phenotyping approaches in any future efforts of this nature.

## Materials and Methods

### Ethics statement

All mouse studies were undertaken by Wellcome Trust Sanger Institute Mouse Genetics Project as part of the International Mouse Phenotyping Consortium and licensed by the UK Home Office in accordance with the Animals (Scientific Procedures) Act 1986. Animal models maintained under the auspices of ethics applications to Monash University were subject to the conditions of the Australian Bureau of Animal Welfare.

### Mouse resources

Mice were generated from ES cells in the KOMP/EUCOMM pipeline and principally compromised conditional ready alleles in which a *lacZ* reporter cassette was integrated upstream of a floxed essential exon [5] (Figure S2). With a few exceptions (n = 24/532) the mice were generated from C57BL/6N ES cells and maintained on this genetic background. A proportion of the lines examined (n = 201), generally those that entered the pipeline in the initial stages of the project, were maintained on a mixed C57BL/6N;C57BL/6Brd-*Tyr^c-Brd^* background. For mice in the histopathology screen details of the strain background, age of sampling, diet, sex, time of tissue harvest, allele name and heterozygosity/homozygosity are detailed in Table S1. Animals were maintained in a specific pathogen free environment with *ad libitum* access to food (typically Mouse Breeders Diet (LabDiets 5021-3, IPS, Richmond, USA)) and water. Specific details of housing and husbandry for individual lines are available from mouseinterest@sanger.ac.uk. For some lines (indicated in Table S1) mice at 4 weeks of age were transferred to a high fat (21.4% fat by crude content; 42% calories provided by fat) dietary challenge (Special Diet Services Western RD 829100, SDS, Witham, UK).

### Additional screen information

All mice generated by the Sanger-MGP underwent a broad primary phenotype screen (“dysmorphology screen”; see Table S1,), which included the visual assessment of skin and hair as described here. Tail skin (n = 2) was collected at necropsy for whole mount immunohistochemistry (IHC) analysis using antibodies against keratins (KRT) 15 and 14 (as per [52] [6]). The activity of the integrated beta-galactosidase (*lacZ*) cassette was assessed using heterozygous cohorts of mice (n = 2 male, 2 female) generated specifically for expression analysis. The pattern of β-gal reporter gene activity was determined by *lacZ* labelling of whole mount tissue preparations from heterozygous knockout. Mice were perfused with fresh cold 4% paraformaldehyde (PFA). Tissues were fixed for a further 30 min in 4% PFA, rinsed in phosphate buffered saline (PBS) and labelled with 0.1% X-gal for 48 hours at 4°C. Samples were post-fixed overnight in 4% PFA at 4°C, cleared with 50% glycerol, transferred to 70% glycerol and imaged.

### Histopathology analysis

Dorsal skin at the thoraco-lumbar region/interscapular region was collected at the time of necropsy, fixed in neutral buffered 10% formalin solution, process routinely, embedded in paraffin, sectioned at 6 µm, stained with hematoxylin and eosin (H&E). Samples from 1–2 female animals were reviewed by an experienced, board certified veterinary anatomic pathologist (JPS). Animals intended for histopathology were reserved for this purpose and not subjected to other phenotyping tests. All slides were scanned and images archived using Zeiss Mirax Slide Scanner and associated software. In addition, representative photomicrographs were taken of all H&E stained slides. Images are available immediately through the Dryad online database (doi:10.5061/dryad.mv34v) and progressively through the International Mouse Phenotyping Consortium website(www.mousephenotype.org) and on the Mouse Genome Informatics webpage (http://www.informatics.jax.org/genes.shtml)(also see Table S1).

### The Sanger Institute Mouse Genetics Project

#### Phenotyping team

David J Adams, Antonella Galli, Amy Gates, Anna-Karin Gerdin, Natasha A Karp, Emma Cambridge, Damian Carragher, Kay Clarke, Jeanne Estabel, Angela Green, Yvette Hooks, Chris Isherwood, Ozama Ismail, Catherine Jones, David Tino Lafont, Chris Lelliott, Simon Maguire, Katherine McGill, Zoe McIntyre, Selina Pearson, Christine Podrini, Hayley J Protheroe, Laura-Anne Roberson, Mark Sanderson, Carl Shannon, Luke Souter, Annie Speak, Agnes Swiatkowska, Elizabeth Tuck, Valerie E Vancollie.

#### Genotyping team

Priya Dalvi, Diane Gleeson, Bishoy Habib, Evelina Miklejewska, Ed Ryder, Debarati Sethi, Sapna Vyas.

#### Mouse informatics

Neha Agrawal, Arthur Evans, David Gannon, Mark Griffiths, Simon Holroyd, Liwen Li, Christian Kipp, David G Melvin, Navis Pretheeba Santhana Raj.

#### Mouse production and distribution team

Joanna R Bottomley, Ellen Brown, Brendan Doe, Evelyn Grau, Nicola Griggs, Richard Houghton, Catherine E Ingle, Helen Kundi, Alla Madich, Stuart Newman, Laila Pearson, Caroline Sinclair, Hannah Wardle-Jones, Sara Valentini, Michael Woods.

#### Mouse facility team

Liam Alexander, Selina Ballantyne, Terry Brown, James N Bussell, Josh Dench, Francesca Flack, Carole Frost, Andrea Kirton, Jordan McDermott, Claire Rogerson, Jennifer Salisbury, Gemma White.

## Supporting Information

Figure S1 Pipelines and phenotype tests (adapted from [1]). (A) MGP phenotyping pipeline shows phenotyping tests and age at which test is undertaken. Red star = skin specific test. (B) Generation of knockouts from the chimera stage to necropsy is shown. Grey = knockout generation strategy, blue = pre-existing/implemented skin screens; white = proposed skin screens.
(TIF)

Figure S2 EUCOMM/KOMP knockout first conditional-ready targeting construct. These lines are predicted to produce null alleles and prediction of gene expression patterns using the integrated B-gal reporter (tm1a (EUCOMM)WTSI)). Conditional (tm1c (EUCOMM)WTSI)) and total knockout (tm1d (EUCOMM)WTSI)) alleles can be generated using *cre* and *flp* mediated recombination.
(TIF)

Figure S3 LacZ reporting of tm1a allele. A) *Aldh18a1* was seen to have broad but weak expression throughout the skin of E14.5 embryos. B) *Krt76* showed expression in suprabasal differentiating keratinocytes of the paw pad epidermis. C) *Nf1* showed expression in sub dermal structures in E14.5 skin. D) *Pfkl1* exhibited strong epidermal expression in E18.5 skin. E) *Rad18* expression was detected weakly in the dermis of E18.5 skin. F) Trpc4ap expression was detected weakly in the dermis of E14.5 skin.
(TIF)

Table S1 Sheet 1 - *Genes and phenotypes identified in gross clinical phenotype and hair follicle cycling screens*. 7 males and 7 female from 362 different transgenic lines were screened for defects in hair follicle cycling and grossly evident skin abnormalities. Sheet 2 - *Genes and phenotypes identified in skin histopathology screen*. Paraffin embedded, H&E stained skin from a cohort including 514 mutants and 44 wild types were assessed for histologic lesions. Sheet 3 - *Genes and MP terms discovered across all tests*. Each screen identified genes that were unique to the specific test, thus increasing the number of genes flagged. Unique MP terms were also described by adding different tests.
(XLSX)

Table S2 Screen parameters. The Sanger-MGP pipeline comprises 263 parameters, with 43 directly relating to the integument. Parameters relating to skin, hair, teeth, and nails are indicated with a 1, whereas non skin parameters are indicated with a 0.
(XLSX)
