# Supplementary figures and images for: Identification of Genes Important for Cutaneous Function Revealed by a Large Scale Reverse Genetic Screen in the Mouse
Source: PLoS Genet. 2014 Oct 23;10(10):e1004705. doi: 10.1371/journal.pgen.1004705 (PMC4207618; doi:10.1371/journal.pgen.1004705)

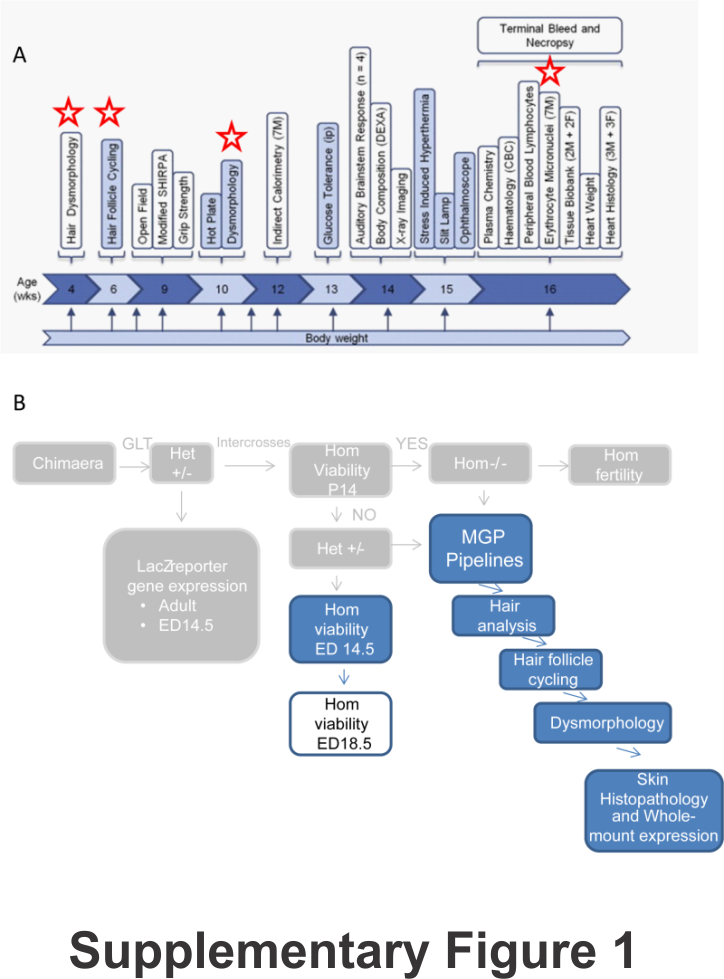

Supplement: Figure S1 — Pipelines and phenotype tests (adapted from [1]). (A) MGP phenotyping pipeline shows phenotyping tests and age at which test is undertaken. Red star = skin specific test. (B) Generation of knockouts from the chimera stage to necropsy is shown. Grey = knockout generation strategy, blue = pre-existing/implemented skin screens; white = proposed skin screens. (TIF) [file pgen.1004705.s001.tif]

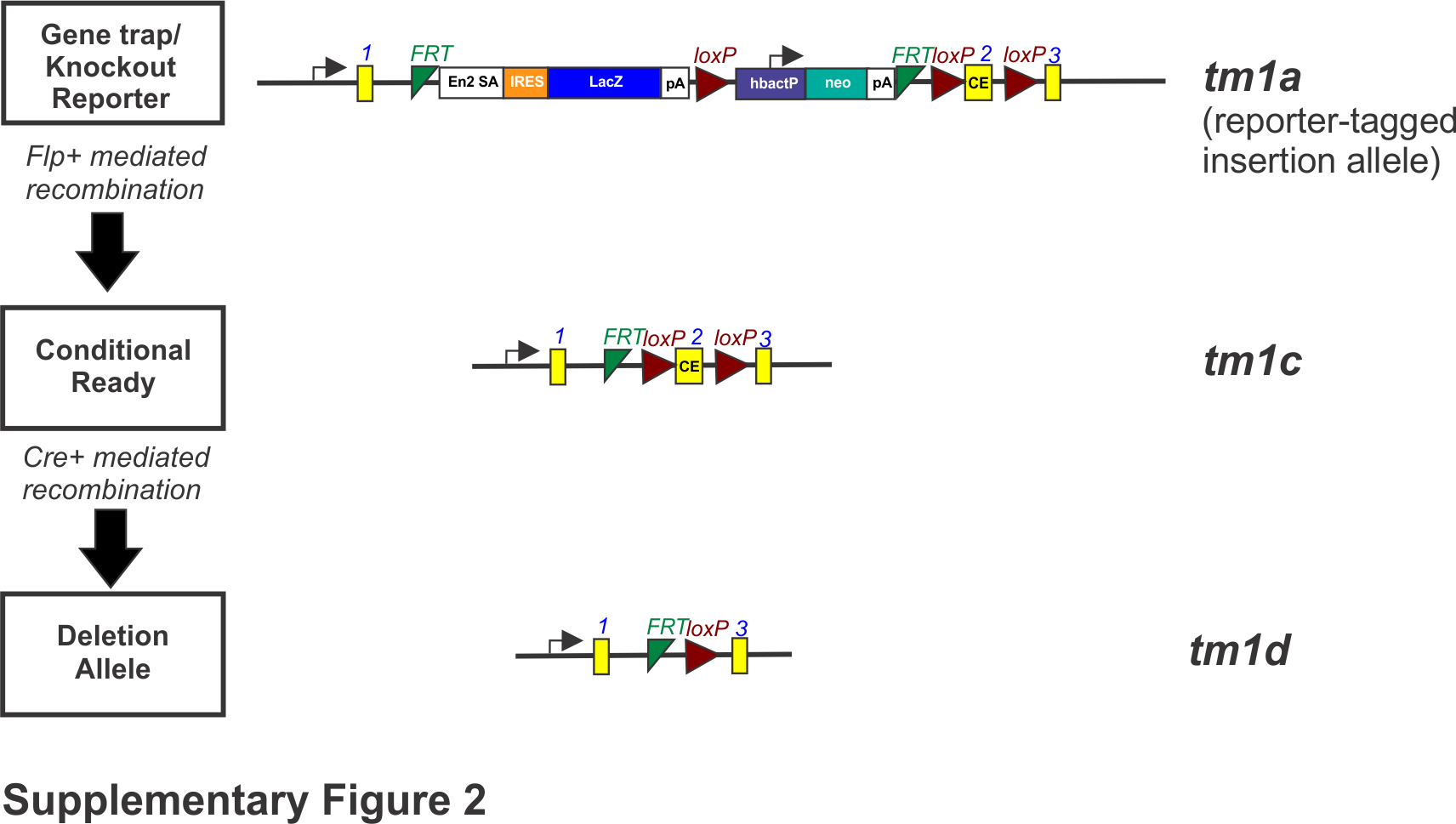

Supplement: Figure S2 — EUCOMM/KOMP knockout first conditional-ready targeting construct. These lines are predicted to produce null alleles and prediction of gene expression patterns using the integrated B-gal reporter (tm1a (EUCOMM)WTSI)). Conditional (tm1c (EUCOMM)WTSI)) and total knockout (tm1d (EUCOMM)WTSI)) alleles can be generated using cre and flp mediated recombination. (TIF) [file pgen.1004705.s002.tif]

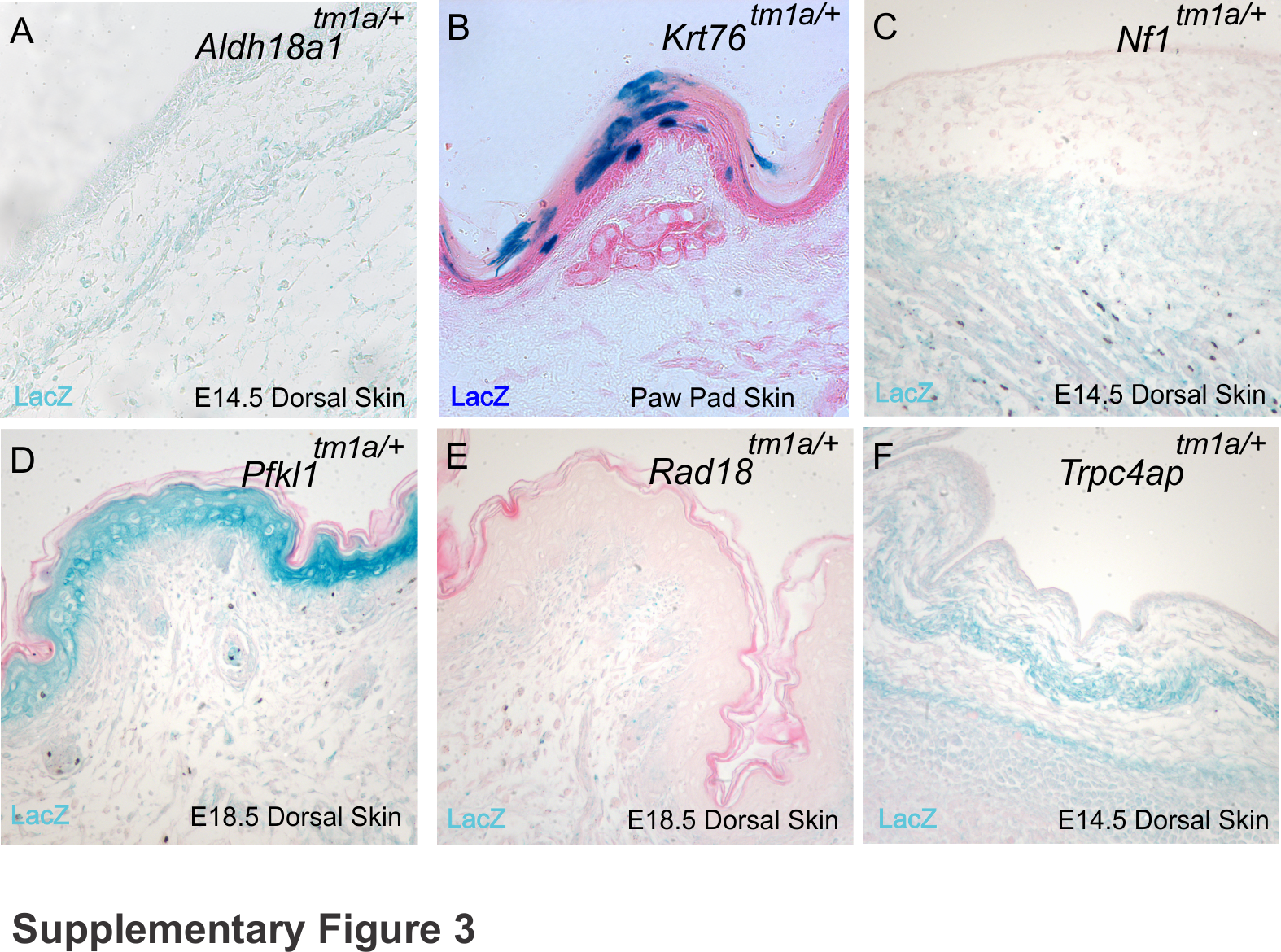

Supplement: Figure S3 — LacZ reporting of tm1a allele. A) Aldh18a1 was seen to have broad but weak expression throughout the skin of E14.5 embryos. B) Krt76 showed expression in suprabasal differentiating keratinocytes of the paw pad epidermis. C) Nf1 showed expression in sub dermal structures in E14.5 skin. D) Pfkl1 exhibited strong epidermal expression in E18.5 skin. E) Rad18 expression was detected weakly in the dermis of E18.5 skin. F) Trpc4ap expression was detected weakly in the dermis of E14.5 skin. (TIF) [file pgen.1004705.s003.tif]
